# A Novel Method to Reduce Time Investment When Processing Videos from Camera Trap Studies

**DOI:** 10.1371/journal.pone.0098881

**Published:** 2014-06-11

**Authors:** Kristijn R. R. Swinnen, Jonas Reijniers, Matteo Breno, Herwig Leirs

**Affiliations:** Evolutionary Ecology Group, Biology Department, University of Antwerp, Antwerpen, Belgium; Panthera, United States of America

## Abstract

Camera traps have proven very useful in ecological, conservation and behavioral research. Camera traps non-invasively record presence and behavior of animals in their natural environment. Since the introduction of digital cameras, large amounts of data can be stored. Unfortunately, processing protocols did not evolve as fast as the technical capabilities of the cameras. We used camera traps to record videos of Eurasian beavers (*Castor fiber*). However, a large number of recordings did not contain the target species, but instead empty recordings or other species (together non-target recordings), making the removal of these recordings unacceptably time consuming. In this paper we propose a method to partially eliminate non-target recordings without having to watch the recordings, in order to reduce workload. Discrimination between recordings of target species and non-target recordings was based on detecting variation (changes in pixel values from frame to frame) in the recordings. Because of the size of the target species, we supposed that recordings with the target species contain on average much more movements than non-target recordings. Two different filter methods were tested and compared. We show that a partial discrimination can be made between target and non-target recordings based on variation in pixel values and that environmental conditions and filter methods influence the amount of non-target recordings that can be identified and discarded. By allowing a loss of 5% to 20% of recordings containing the target species, in ideal circumstances, 53% to 76% of non-target recordings can be identified and discarded. We conclude that adding an extra processing step in the camera trap protocol can result in large time savings. Since we are convinced that the use of camera traps will become increasingly important in the future, this filter method can benefit many researchers, using it in different contexts across the globe, on both videos and photographs.

## Introduction

Camera traps, triggered by motion and/or heat of a passing subject, are a non-invasive way to study animals and their behavior. Valuable knowledge is gathered by registering animals in their natural habitat. Questions addressed by using camera traps are often related to animal ecology, behavior and conservation [1]. For example, camera traps have been used to study niche separation [2], competitive exclusion [3], population structure [4], [5], density estimation with [6], [7] and without individual recognition [8], [9], abundance estimation [10], foraging behavior [11], biodiversity [12], activity patterns [13] and habitat use [14], [15]. Camera traps can replace other study methods or add to direct observations, track inventories, knowledge of local inhabitants or genetic surveys [16]–[20]. The target species are mostly medium to large terrestrial mammals since capture probability decreases with decreasing size of the species [21], [22]. In recent years, species such as small arboreal primates [23], [24], ectothermic Komodo dragons [25] and birds [26], [27] have been subjects of camera trap study, showing wide ranging applicability.

Rovero et al. (2013) showed that the amount of camera trap papers being published in biological research is still increasing [28]. Although a number of papers are published concerning methodology (see above), data processing, the step between the downloading of the images and the start of the statistical processing of data obtained from the images, is often overlooked. The switch from analog to digital cameras and technical progress such as increasing data storage capacity, caused the amount of recordings to increase massively. As a consequence, processing recordings became more time consuming and is becoming one of the limiting factors in the use of camera traps [29]. Automated image analysis has been used in different biological contexts ranging from microbiological ecology [30], experimental laboratory systems [31], phenotypic analysis [32], remote sensing [33] to corridor mapping [34]. However, publications about software solutions to manage and analyze image data and metadata collected by camera traps are limited [29], [35]. The TEAM (Tropical Ecology Assessment & Monitoring) network uses a software package to manage and standardize image processing since they expect to have 1 000 000 photographs per year [36]. Yu *et al.* (2013) recently proposed a method to automatically recognize species, based on manually cropped camera trap photos of animals, however, for their algorithm to work, they still had to visually inspect and manually select all recordings [37]. The method proposed in this paper addresses the previous step in the workflow, namely reducing the amount of recordings that must be visually inspected by automatically classifying the recordings according to the potential of containing the target species (see Material and methods).

Although pictures are more commonly used in camera trap studies and are easier to process, videos provide more detailed information, especially behavioral. They are used in determining competitive exclusion [3], observing time budget [38], observing behavior and determining population structure [4], [39] and to study nest predation [40]. Videos are also more appealing to the general public when used as an awareness tool. Technological advances and continued innovation will ensure that camera traps will play an increasingly important role in wildlife studies [1]. We expect that the use of videos will increase and so will the need for a tool to automatically identify the non-target recordings.

In this study, we develop such a tool based on recordings gathered while studying beavers. We used camera traps to record the spread of recently reintroduced beavers in northern Belgium, to evaluate territory occupancy by individuals or mated pairs and to study activity patterns.

Our first results indicate that, although the target species was recorded frequently, the majority of the recordings were empty or contained non-target species. Since it is very time consuming to watch all recordings, we developed an automated filtering method. The goal of this algorithm is to eliminate the maximum amount of the non-target recordings while minimizing the amount of target recordings discarded. To our knowledge, this extra filtering of recordings (photos or videos) between the downloading of the recordings and the manually processing was never reported before in camera trap studies.

## Materials and Methods

Bushnell Trophy cams (Bushnell Outdoor Products, 9200 Cody, Overland Park, Kansas 66214, Model 119436c) were positioned at 12 different locations in 9 different beaver territories, in the province of Limburg, in the east of Flanders, 20 July 2012 - 8 October 2012. The responsible authority, the ‘Agentschap voor Natuur en Bos’ (Agency for Nature and Forest) decided that, although beavers are a protected species, no special permit was required since camera traps do not disturb the animals. Permissions to access the camera trap locations were granted by Limburgs Landschap (1 location), Natuurpunt (5 locations), nv De Scheepvaart (1 location) and Steengoed (5 locations).

Cameras were attached to a nearby tree 30–90 cm above the ground and directed at the burrow (5 locations), a running track (3 locations), or a foraging location (4 locations). The anticipated passage of a beaver was never farther than 6 m away from the camera (but often closer). The camera settings were standardized over all cameras as follows: Type = Video, Video Size = 720×480, Video Length = 15s, Interval = 1s, Time Stamp = On, Video sound = On. Cameras were activated when detecting a combination of movement and heat. Cameras were positioned for an average of 35 days at each location (range = 30 to 50 days, SD = 5.6). The sensitivity of the sensor was set to low, medium or high, according to local circumstances. The medium sensitivity was used in most environmental settings. When cameras were directed to highly dynamic streams, the sensitivity was set low. When beavers were expected to pass rather far from the camera and vegetation was limited, high sensitivity was selected. The sensitivity must be sufficiently high since we suspect that the cold water in the fur of the beavers reduces the probability of detection by camera traps. The illumination of recordings in poor light conditions (dusk, dawn or night) was assisted by infrared LEDs, resulting in black and white recordings. Only black and white recordings were considered since it is known that beavers are nocturnal [41]. All movies were saved in the.AVI format on a Transcend SD HC 16GB card, and copied in the field to a small portable computer to a unique folder per camera and location. Filename, date and hour of the recordings were automatically extracted to Excel. Location, camera number and species were manually entered. An extra category was added cataloging image as ‘beaver’ or ‘non-target’ (including empty images and recordings of other animals) since our main interest was to separate beaver recordings from all the rest.

Camera locations were divided according to the area of water visible in the video. The area was measured by using ImageJ, an open source application to process images [42]. When the surface of the water was <10% of the frame, videos were classified as Dry (5 locations, water surface ranges from 0–4%), while locations with >10% of water surface in the recordings were classified as Wet (5 locations, water surface ranges from 12%–57%). Two locations varied because of rainfall and drying of the water body respectively between 0% and 48% and 0% and 53%, and were included in the Wet classification since not enough beavers (n = 15) were registered to analyze these locations separately.

We were ultimately interested in recordings of beavers. But using computer algorithms to discriminate beavers in video footage was a very difficult endeavor, requiring high-level pattern recognition, which was by no means the aim of this study. Our goal was less ambitious, as we tried to discriminate the videos that were likely to be beaver free (non-target recordings) and to reduce the set of videos that had to be inspected for beaver presence. As a consequence, we tried to maximize the true positive rate (TP-rate; number of non-target recordings correctly classified as non-target recordings divided by total number of non-target recordings) while minimizing the false positive rate (FP-rate; number of target recordings wrongly classified as non-target recordings divided by total number of target recordings) (Table 1).

**Table 1:** Classification matrix of the recordings.

		Reality	
		Non-target recordings	Target recordings
Classification result	Non-Target recordings	True Positive	False Positive
	Target recordings	False Negative	True Negative

1991 videos were recorded at 12 different locations in 9 different beaver territories, in the province of Limburg, in the east of Flanders, Belgium, between 20 July 2012 and 8 October 2012. We recorded 1043 recordings of the target species, the beaver, 553 empty recordings and 395 recordings of non-target species. Every recording was classified based on *D* = “the amount of pixel variation” as target or non-target recording. The correct classification of non-target recordings was considered to be a success (True positive, TP) since these recordings can be correctly discarded. This value must be as high as possible in order to remove the maximum amount of non-target recordings. False positives (FP) were the beavers (target recordings) which were classified as non-target recordings and wrongly discarded. This number must be as small as possible since valuable data is being discarded. False negatives (FN) were non-target recordings which were classified as being target recordings. True negatives (TN) were the target recordings which were recognized as being target recordings.

The discrimination was based on to what extent the video frames change throughout the length of the video. As beavers are fairly large mammals (0.80–1.20 m body length, 0.25–0.50 m tail length, 11–30 kg [43]) and among the largest in our study area, it is to be expected that their presence on the footage will induce bigger changes, compared to other smaller animals, e.g. small rodents and birds, or movement of water and/or vegetation registered in the recordings. In the following, we propose and evaluate two different ways to quantify the amount of ‘movements’, on which the discrimination will be based. All following manipulations and calculations were performed in MATLAB [44] except when stated differently.

To start, we performed two basic manipulations of the recordings. The first two frames were removed from each movie because of the time stamp on the first frame and the instability of the light caused by the starting of the camera in the first two frames. Second, we averaged out small spatial and temporal changes due to detection noise, movement of vegetation, water reflections, etc. This was done by down sampling (averaging) the video along both the spatial, x and y, and temporal dimension, t (respectively with factor 5, 5, 10). As beavers are large and move rather slowly, the detection of their movement would not suffer from this reduction. Moreover, this operation would also improve computational speed.

To detect non-target recordings, we first quantified the amount of variation throughout the video. If the pixel values did not vary much throughout the video, then it was unlikely that the camera was triggered by an animal as large as a beaver. We used two different methods to quantify this amount of pixel variation and arrived at a ‘measure of frame variation’. Both were chosen since they are easy to apply and differ qualitatively from each other.

In the first method, Filter 1, we considered the frame variations compared to the average frame 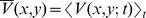, of which every pixel (x,y) was averaged across the length of the video (along t = time). At every time step the squared distance of the frame 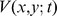 to the average frame is calculated, 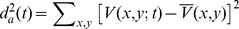.

We used the following equation as a measure of the ‘amount of pixel variation’: 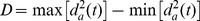.

Note that for the calculation of *D* we subtracted 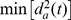 in order to compensate for the fact that different environments show different base line variations, e.g. aquatic sceneries exhibit more variations compared to dry area and we were interested in the movements of animals against this variable background.

The second method, Filter 2, focused on the changes between subsequent frames 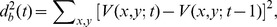
and is essentially different from the previous method. The parameter *D* was calculated in the same way as before (see Matlab Code S1).

Both methods quantify the changes in pixel values within the recording, which we consider to be a proxy for movement. If this *D* value is fairly low, then the recorded movements were rather small; a larger value points to increased activity during the 15 second recording. These calculated values were used to discriminate the assumed non-target recordings from the possible target recordings.

This discrimination was done by means of a threshold: if the calculated *D*-value of the video was below this threshold, then we assumed that the video was empty (a non-target recording); we did not make any inferences on the target’s presence when the threshold value was exceeded. Hence, our approach was aimed at detecting non-target recordings. Classifying a video without a beaver (empty or with other species) as a non-target video was considered a success, see Table 1. Consequently, for a particular threshold value, one could calculate the true positive rate (TP-rate; the number of correctly classified non-target recordings divided by the total number of non-target recordings) and the false positive rate (FP-rate; the number of beaver recordings misclassified as non-target, divided by the total number of beaver recordings) that a classification based on this threshold would produce. Variation of the threshold over the full range of D-values, resulted in the so-called receiver operating characteristics curve (ROC curve, [45]), from which the costs and benefits corresponding to a particular threshold could be easily assessed. This analysis was performed in RStudio [46].

We also calculated the time savings generated by using a filter method, as the summed duration of the recordings that would not have to be inspected visually. To determine the ability of the threshold to discard the intended amount of non-target videos in spite of sampling variation (i.e. proportion of beavers discarded in future videos), a bootstrap procedure was carried out. Briefly, a subset of 500 observations were resampled from the dataset and classified according to the original 5% FP-rate. The operation was repeated 1000 times and the FP-ratio was calculated at each step. Descriptive statics for the bootstrapped FP-ratio were computed for the Complete, Dry and Wet datasets.

## Results

**Figure 1:**
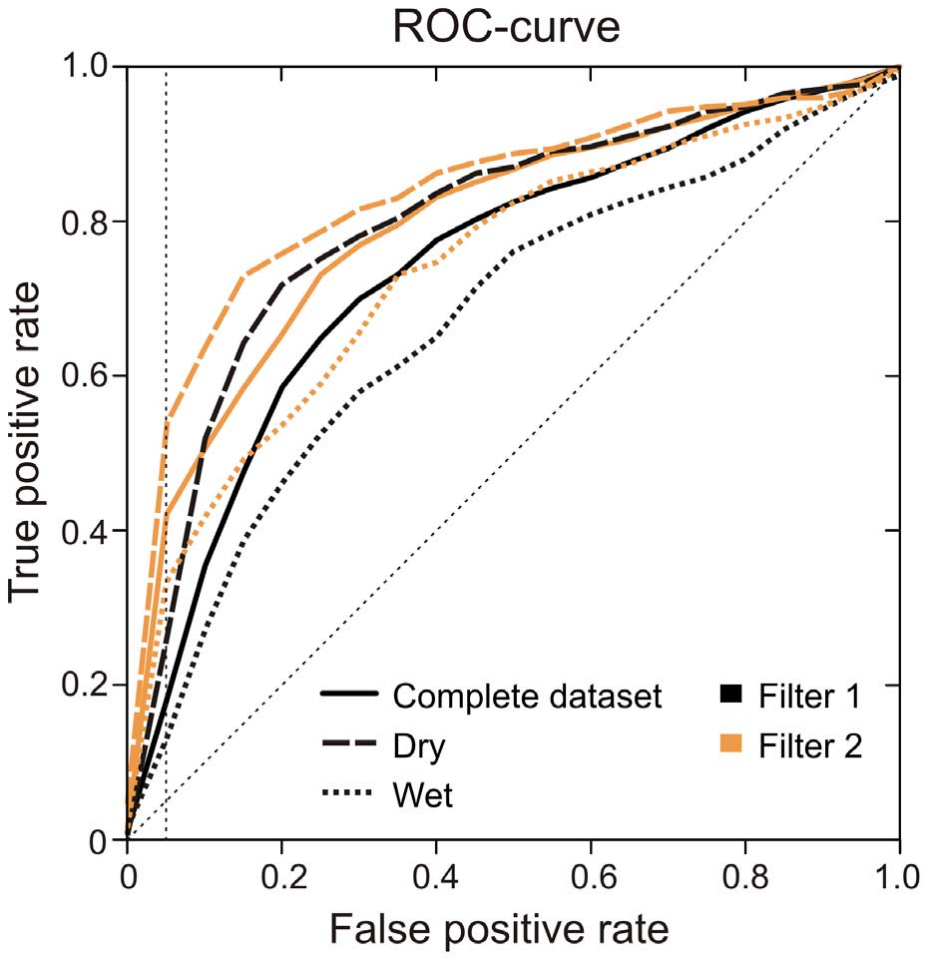
Possible gain (true positive rate, TP-rate) given an accepted loss (false positive rate, FP-rate). The FP-rate represents the proportion of target recordings (beavers) classified as non-target recordings. The TP-rate is the proportion of non-target recordings correctly classified as non-target. This is the proportion of non-target recordings that will be discarded correctly given a certain FP-rate. The best performing filter maximizes the TP-rate while minimizing the FP-rate. Filter 2 performs better in all environmental circumstances. The dashed diagonal represents the outcome of a random model which cannot discriminate between target and non-target recordings. The dashed vertical line represents a 5% threshold (FP-rate). Dry<10% water area in footage (5 locations, n = 933 recordings), Wet>10% water area (7 locations, n = 1058 recordings), Complete dataset is the combined Dry and Wet dataset (12 locations, n = 1991 recordings).

During 405 camera nights, the 12 cameras recorded 1991 videos, 933 recordings in dry locations and 1058 in wet locations, with a mean of 166 recordings per location (49–296, SD = 81.9). All recordings were watched and cameras registered 1043 recordings of the target species, the beaver, 553 empty recordings and 395 recordings of non-target species. The resulting ROC-curves are shown in Figure 1 for the two different filters, when applying them to videos recorded in different conditions. Both filter methods discriminated between non-target and potential target recordings, but Filter 2 performed slightly better than Filter 1, especially when lower FP-rates were tolerated (5% FP-rate marked by a vertical dashed line). Also, irrespective of the FP-rate, the TP-rate in Dry circumstances was always higher compared to Wet circumstances.

The FP-rate that can be tolerated depends on the study goals and design, and will be most decisive in what threshold can be used. In this particular study, a loss of 5% to 20% of the beaver footage was tolerable. A FP-rate of 5% resulted in a reduction of workload allowing us to remove 26% to 53% of non-target recordings (time savings 30–53 min) in dry conditions, 13% to 33% (time savings 25–55 min) in wet conditions and 18% to 42% (time savings 56–113 min) in the complete dataset. When we tolerated a FP-rate of 20%, then 72% to 76% (time savings 92–95 min) of non-target recordings could be discarded in dry conditions, but only 46% to 54% (time savings 92–104 min) in wet conditions and between 59% and 65% (time savings 191–206 min) in the complete dataset.

The bootstrap analysis indicated that sampling variation had a limited effect on the FP-ratio (Table 2). Within the 95% confidence interval, a minimum of 2.9% and a maximum of 7.4% of beaver images would be discarded using the 5% threshold, based on the complete dataset. Results of the bootstrap analysis indicated that sampling variation had only a limited effect in all environmental circumstances on the percentage of recordings discarded.

**Table 2:** A bootstrap analysis was performed for both filter methods (Filter 1 and Filter 2) on the complete dataset (n = 1991), on the videos recorded at dry locations (n = 933) and on the videos recorded at wet locations (n = 1058).

		Dry		Wet		Complete dataset	
		Filter 1	Filter 2	Filter 1	Filter 2	Filter 1	Filter 2
False Positive Rate	Mean	0.051	0.051	0.050	0.051	0.051	0.051
	Sd	0.009	0.009	0.011	0.010	0.012	0.011
	2.5 percentile	0.035	0.035	0.029	0.032	0.029	0.029
	97.5 percentile	0.068	0.067	0.072	0.072	0.074	0.074

A subsample of 500 videos was randomly sampled with replacement and this was repeated 1000 times. Recordings were classified based on their *D*-value; the thresholds were chosen to result in a 5% false positive rate in the respective datasets (see text). The new mean, standard deviation (Sd), 2.5 and 97.5 percentile are shown.

## Discussion

We show that a filter method based on changing pixel values can partly discriminate between recordings of the study species and non-target recordings. The amount of non-target recordings that can be discarded without watching the footage depends on the chosen threshold and this threshold will vary between studies.

In both filter methods, it is clear that results depend on the environmental circumstances of the camera locations. It is easier to distinguish between beaver and non-target recordings in Dry circumstances because variation is lower than in Wet circumstances. The filtering mechanism will work best on medium to large mammals, but these are also the most suitable subjects of species inventory studies by camera traps [47]. Since these filter methods are the first attempt to partially automatically process recordings, we did not aim to create a very complicated high level pattern recognition program. The current method is rather robust to changes in camera-angle, illumination, colour or black and white recordings, distance from the camera to the subject, size of the subject and the position of the subject to the camera since they can be largely accounted for by choice of the threshold.

The greatest differences between filter methods were observed when the tolerated FP-rate was small. When using a FP-rate of 5%, Filter 2 resulted in a TP–rate which was more than double the value of Filter 1, in all environmental circumstances. When increasing the tolerated FP-rate, these differences became smaller (Figure 1).

Discarding up to 76% of non-target images can prove to be very timesaving when used in long term survey studies deploying large number of cameras. This reduction of workload has two direct implications for camera trap studies. Since the processing of recordings takes less time, the number of cameras can be increased allowing covering a larger study area or augmenting the amount of cameras used in the same study area so that patterns can be discerned on a finer scale. Second, the sensitivity of the camera traps can be increased, allowing the cameras to react to smaller animals. This will result in an increase of the number of recordings, but the time spent on processing the images will still be reduced because of the filtering of non-target recordings. The recordings of small (non-target) animals may be useful for other studies. The same database of video recordings could then be used, but now with the small animal as target, without having to perform a new field campaign.

Although we show that the proposed filter method can reduce the amount of work substantially given a certain cost (lost of recordings of interest), there are limitations to this method.

First, a number of recordings must be viewed to determine if the species of interest is recorded. Only when a sufficient number of recordings of the species of interest is obtained, a comparison between these and other images can be made. The necessary amount of recordings depends highly on the studied species (size and speed), other sympatric species and the environmental conditions. Larger species that are detected regularly and will be studied for a long time by using camera traps are the most suitable subjects to discriminate from non-target recordings. Based on the determined threshold, images which most likely not contain animals can be filtered out, but a distinction between different species of similar size and speed is not feasible.

The filter methods are based on changing pixel values. Animals that do not move (stay on the same location during the length of the complete recording) will most likely be discarded. It is however reasonable to assume that a motionless animal was detected while arriving or will be detected while leaving the location where the camera trap is aimed at. Animals must be of sufficient size to stand out from the background noise and to be recognized as a potential target recording. For very small species, it may be necessary to skip the down sampling step in both spatial directions in order to achieve an image that is detailed enough. For very fast species, the down sampling in the temporal dimension can be detrimental. However, the performance of the method depends highly on the variation (movement) in the background. Once the variation in the background is larger than the changes induced by passing species, it will not be possible to distinguish between target and non-target recordings.

The use of the filter protocol must be considered with knowledge of the species, species community and the study design. For example: when camera traps are used to perform capture mark recapture analysis on individually recognizable individuals [4], [48], [49] it is probably not acceptable to miss a recording since a single recording may have a large impact on results. For this study, a loss of 5% to 20% of the beaver footage was tolerable, since beavers were recorded frequently and the data were collected to determine average activity patterns. A reduction of the tolerated loss of target recordings would have lowered the TP-rate, resulting in less time savings. How quickly the TP-rate decreases with decreasing FP-rate depends on the shape of the ROC-curve and will differ between studies. Also, these filter methods are not suitable for species which are very hard to record because it will take a very long time before enough data are collected to determine the threshold.

The filter protocol is optimized for processing videos and not photographs. The same process could be used for processing photographs. Although each picture is a still image, the comparison of consecutive pictures (as if they were frames of a movie) makes the analysis possible. The disadvantage of this method is that the time between pictures can vary a lot when cameras are movement-triggered, and environmental factors, like growth of plants or changing light conditions, can make it more difficult to compare different photographs. However, Hamel *et al.* (2013) showed that using a fixed 5-min interval resulted in lower daily presence raw error rates compared to movement-triggered cameras and recommend opting for time-triggered cameras when aiming to capture abundant species [50]. When using fixed time intervals, high variation is avoided, making it easier to process images when the interval between pictures is not too long. However, problems can still occur when light conditions change rapidly as during sunset and sunrise. When using a series of pictures that are taken with fast intervals, as a consequence of a single trigger event, this problem can be avoided.

Camera traps are imperfect detectors and the chance of detecting a species decreases with decreasing species size and increasing speed and is influenced by seasons [22], [51]. But a camera can also be ‘tricked’ into recording when there are no animals. This generates empty recordings and these are rarely reported although they give an important indication about the efficiency and expected time effort necessary to process all recordings. The reasons for recording empty recordings are diverse, moving vegetation, technical problems, moving water, sunlight reflection or time lag between passing of an animal and starting of the camera. These factors will vary depending on environment, season and study species. These problems are well known to researchers but receive only limited attention in the literature [52]. To reduce the amount of empty recordings, the sensitivity of the sensor can be reduced. However, this also increases the chance of missing the study species. Although no published research is available, we think that sensor sensitivity must be augmented in semi-aquatic mammals when being filmed on land since they are likely to just have left the water, resulting in a cold layer around the body of the animal making it more difficult to detect than a similar sized dry mammal. This high sensitivity results in more false detections, explaining the high percentage of empty recordings compared to other studies 15,7% [20]. We want to encourage authors to not only mention the trapping effort and the amount of pictures of the studied species but also the amount of empty recordings (or recordings of non-target species) making it possible to compare how different types of cameras perform in different circumstances allowing future researchers to benefit from this knowledge.

Although we acknowledge the limitations of our filter method, we believe an important progress has been made by showing that adding an extra filter step between downloading of images and the statistical processing can save lots of valuable time, with losing only a limited amount of data.

## Supporting Information

Code S1 **Matlab Code.**
(M) Click here for additional data file.
